# A Critical Interpretive Literature Review of Phronesis in Medicine

**DOI:** 10.1093/jmp/jhae045

**Published:** 2025-02-19

**Authors:** Sabena Yasmin Jameel

**Affiliations:** University of Birmingham Medical School, Birmingham, United Kingdom

**Keywords:** *Aristotle*, *phronesis*, *professionalism*, *virtue*

## Abstract

This article presents the results of a rigorous critical interpretive review that maps the current literature on phronesis in medicine. The literature in this area involves varied disciplines, centuries, and conceptions and is extensive, but through a focused review, this study seeks to clarify definitions and key distinctions. It thereby aims to elucidate a depth of meaning and understanding regarding phronesis in medicine to inform future work on the topic. Specifically, 12 themes are inductively identified and analyzed from the literature, and organized into three chronological categories of past, present, and future. A narrative summation of the literature to date is then offered, assessing the varied conceptual applications of phronesis to medical practice, the emerging literature on its applicability to organizations, prospects for empirical work on the concept, and its application in medical education.

## I. INTRODUCTION

Rooted in the virtue ethics tradition, *phronesis* (practical wisdom) is a morally grounded, action-orientated master virtue, which adjudicates when values conflict. Aristotle describes phronesis as an intellectual virtue with an executive function, and in the *Nicomachean Ethics*, he suggests it can be taught and learned. Phronesis operates on the level of particular contextual decisions rather than on the generalized, often reductive levels of evidence-based medicine (EBM). Phronesis is neither simply cleverness (*deinotes*) nor cunning (*metis*). For Aristotle, phronesis is a route to eudaimonia (flourishing), such that a central notion in the concept is “*all things considered, in relation to human flourishing.*” For a clinical *phronimos* (a practically wise doctor), the evidence is incorporated into a deliberative, morally orientated contextual outcome.

The exponential increase in medical evidence pitched against ever-complex social, economic, and technological contexts often brings value conflicts to the fore, and it is easy to see how understanding phronesis could illuminate medical education and practice. A revival of phronesis in medicine was initiated by Pellegrino and Thomasma in their seminal book *The Virtues in Medical Practice* (1993). The term is increasingly being studied in the academic literature today, but up until the turn of this century, it remained largely unexplored. Moreover, most of the published work to date is theoretical in nature, which invites an opportunity to perform empirical work on the concept and its application.

In this article, I outline how a critical interpretive literature review was conducted to map the literature on *phronesis in medicine*. The review is tightly focused and seeks to clarify definitions and elucidate the depth of meaning in the understanding of phronesis in medicine, enabling future work to grasp what preceded it. This literature appraisal shows that phronesis is understood in different ways by different authors, and in this context, there is a need to identify conceptual boundaries and a meaning and translation of the concept relevant to everyday medical practitioners. The narrative that accompanies the literature summary below therefore aims to assist in the conceptualization of practical wisdom, helping to guide a meaningful integration of caring, competence, and character development required for the practice of medicine.

## II. A CRITICAL INTERPRETIVE LITERATURE REVIEW

The literature review is a methodological synthesis of a scoping review and the critical interpretive method, which provided rigorous, transparent, and reproducible methods of mapping the literature (Arksey and O’Malley, 2005; McDougall, 2015). The literature in this area is mostly theoretical (normative and evaluative), which does not lend itself well to formulaic quality assessment seen in systematic reviews (McDougall, 2015).

Three research questions were formulated and addressed in the literature review:

1. What has been written about Phronesis in Medicine?
2. What empirical research has been done on Phronesis in Medicine?
3. What narrative commentary prevails in relation to Phronesis and Medicine?

### Identifying Relevant Papers

An Ovid Medline search was performed in 2020 looking for papers from 1946 to February 2020 (the longest period of search at the time) in the English language. Most literature in this field appeared after 1986. The exact search terms Phronesis AND Medic* were used again on other databases (Web of Science, Ovid ASA Psychinfo, EMBASE, CINAHL, and ASSIA), and later expanded to include the terms Practical Wisdom AND Medic*. All relevant papers were read in full. A more recent 2023 update on the original search has been incorporated into this article.

The prefix Medic* was used to capture medicine, medical practice, medical education, medical competence, medical decision-making, medical judgement, and medical speciality across medical disciplines. The original scoping review did not include the terms Clinic* and Prudence/Pruden*, but these could be incorporated in a future study.

**Fig. 1.**
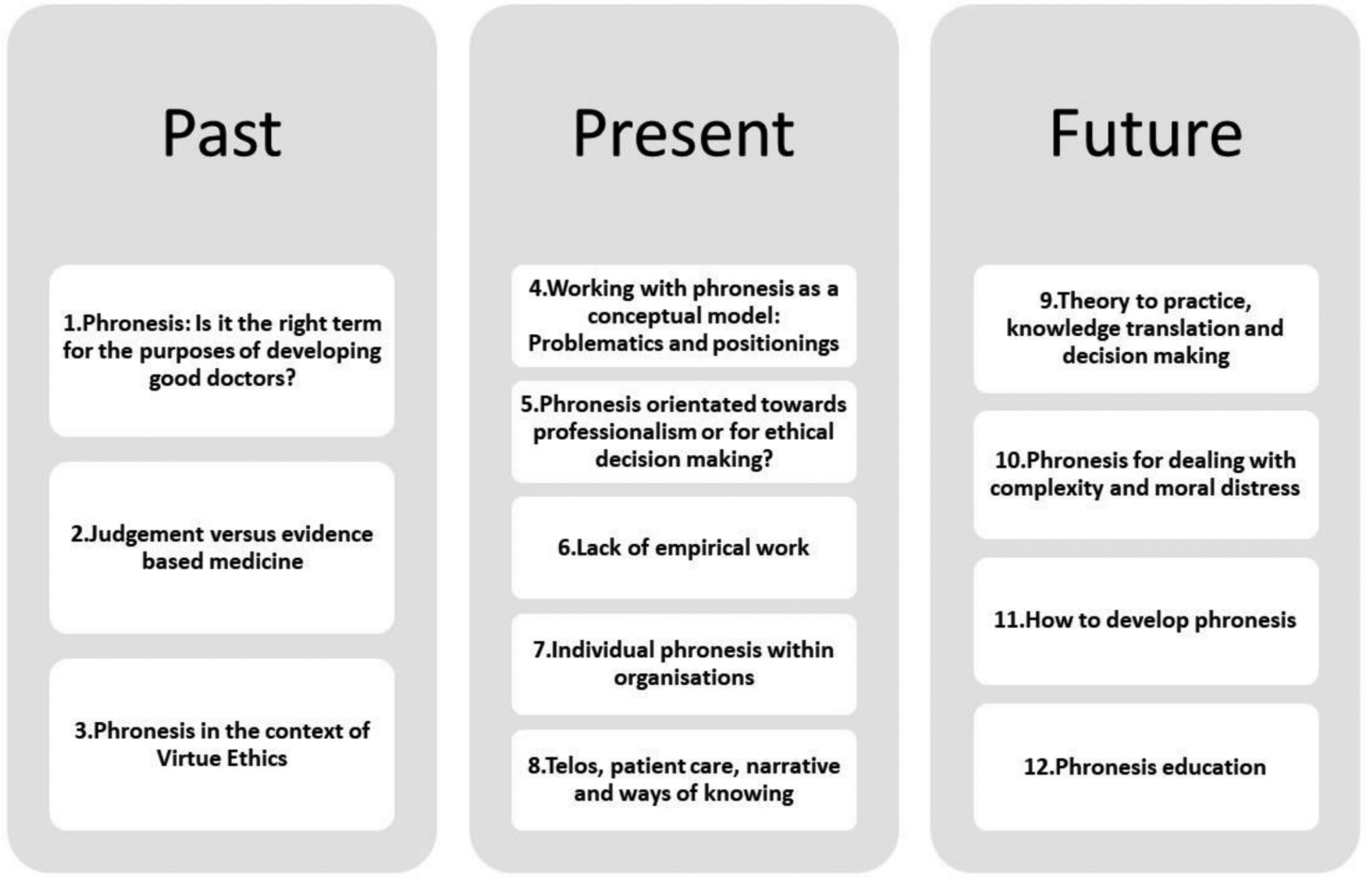
The 12 literature review themes.

From the 73 papers that were found to be relevant, 12 themes were distilled and have been summarized in narrative form below. The 12 themes have also been organized into three chronological categories (fig. 1), and a summary statement of each theme is collated in table 1. This article does not tackle theme 3 due to space limitations and because articles in this special issue of the journal already contain comprehensive insights from moral philosophers who expertly outline phronesis in the context of virtue ethics (Fowers et al., 2024).

**Table 1.** Summary statement of themes.

***Theme 1*** The literature supports the assertion that phronesis is the right term for developing good doctors, despite undergoing an evolution from Aristotle’s original thoughts. ***Theme 2*** The literature argues that phronesis has superior qualities in relation to clinical decision-making when compared to the application of technical rationality demonstrated in evidence-based practice. ***Theme 3*** The literature asserts that rules-based ethics, which dominate healthcare practices (deontology and utilitarianism) are insufficient in medical practice because of their abstraction when applied to patient contexts and medical complexity. Virtue Ethics, in particular, the intellectual virtue of phronesis, is a better heuristic for modeling good physicians. ***Theme 4*** Kristjánsson (2016) offers a paper that allows clinical academics to reflexively analyze how they conceive phronesis in terms of four binaries. These binaries illuminate the stance a researcher takes, or how educators position themselves when educating for phronesis. Making these positions explicit offers clarity from assumptions. ***Theme 5*** The literature positions phronesis as a process that encompasses medical professionalism rather than just the ethical aspects of practice. ***Theme 6*** Up until 2019, there was little empirical work on phronesis in medicine, with most literature being theoretical. There has been a subsequent upturn in published work, allowing a more nuanced grasp of phronesis in practice. ***Theme 7*** In *Nicomachean Ethics*, Aristotle focuses on phronesis in the individual. Most of the published literature on phronesis in medicine focuses on individual phronesis. Some literature begins to explore the concept of collective phronesis, building on Aristotle’s *Politics* and outlining the importance of an organizational perspective. ***Theme 8*** To encourage phronesis, bioscientific positivistic knowledge is not sufficient; it should be balanced with more subjective and interpretive ways of knowing, such as promoting better ways to understand the self and others. Interpreting narrative and the use of medical humanities are useful educational tools. ***Theme 9*** Phronesis means being practically wise. Literature has examined how the theory is put into practice, i.e., the enactment of wise medical practice, and it also suggests that the current infrastructure in health care is inflexible. ***Theme 10*** Some literature has highlighted the value of phronesis in dealing with complexity and moral distress experienced in medical practice. ***Theme 11*** Significant literature has described how a curricular and organizational focus on aspects such as moral development, goals of medicine, self-awareness, emotional regulation, reflective practice, mentorship, role models, and understanding narrative can help develop phronesis. Numerous erosive factors have also been identified. However, a key question remains debated: whether phronesis can actually be taught or if this pedagogy is more a case of providing the environment in which it can develop. ***Theme 12*** The past 30 years have enabled progressive debate about the constituent elements of phronesis in medical education. This then allows discussion about factors that hinder and those that support its development. Important elements have been identified and these include the goals of medicine and its moral underpinnings, communicating the language of virtue literacy, embedding the concepts in curricula and the role of assessment.

## III. THE PAST

### Theme 1—Phronesis: Is It the Right Term for the Purposes of Developing Good Doctors?

Aristotle describes phronesis as the ability to apply universal or general knowledge to particular situations. The *phronimos* identifies what is unique and particular about the situation, making a moral judgment about the resultant action, and in so doing, *eudaimonia* (flourishing) is the expected result or goal. With respect to medicine, Aristotle (2004) described the practice as a *technē* (skill/technical expertise), with the goal being good health [NE VI, 1094a 9]. However, he also used medicine as an example of phronesis (Kristjánsson, 2015), and this ambiguity in Aristotle has been the focus of academic deliberation. The literature review supports a lack of clarity in the application of terms like *technē* (craft/skill), *episteme* (scientific knowledge), *sophia* (theoretical wisdom), *nous* (intellectual insight), *praxis* (practice), *poesis* (creation of something new), and *phronesis* (practical wisdom) in relation to medical practice (and in relation to each other).

The literature review identified that much of the literature refers to medicine as praxis, but also as technē. Svenaeus (2003) states that medical practice is not the same as praxis, but that phronesis is the best way to capture what is required. Joseph Dunne (2009) alludes to the difference between technē (doing) and phronesis (being) and explains why he feels medicine is not merely about phronesis. The perceived dichotomy between technē and phronesis, and Dunne’s view, has been analyzed in detail by Kristjánsson (2016).

More recent literature, however, suggests that phronesis is a more appropriate knowledge process than technē in the realms of contemporary medical reasoning. However, it could be that the goals of medicine (telos) determine whether it is a praxis or technē. If the goal is *healing of illness*, which considers the psychological processes required to feel subjectively well, then medicine would be a praxis that is ethically directed towards flourishing. Compare this to the goal of the medical *treatment of disease,* which would make medicine more of a technē, considering the body as a biomedical machine that can be fixed. The literature review demonstrates there is no consensus regarding the telos of modern medicine, hence, the ambiguity in defining it as praxis or technē.

Edmund Pellegrino mentioned the importance of Phronesis in Medicine in 1979, further developing these thoughts in a publication with Thomasma, *The Virtues in Medical Practice* (1993). In Chapter 7 of that work, they claim that phronesis, which coordinates all the different moral virtues, is “indispensable” for the wise moral action of the good doctor. Gatens-Robinson (1986) was also an early advocate for phronesis being a useful model for the rational orientation of clinical judgment in medicine. He argued that medicine is a human science and requires interpretation and judgment which is best captured by phronesis. In 1997, Davis anchors his argument on Pelligrino’s philosophy of medicine as healing (rather than fixing), and argues that the most compelling paradigm for the rationality of the physician’s actions is phronesis.

In 1999, in a pediatric surgery journal, Hutson and Myers presented a very real clinical dilemma for surgeons dealing with severely disabled children and offered phronesis as a framework for exploring the complexity of the situation. They suggested that with increasing medical and technical advances, ethical considerations should be part of the decision-making process (Hutson and Myers, 1999). Tyreman (2000) then published aligned thoughts, suggesting that uncertainty is an innate feature of clinical practice and largely due to the interpretative nature of illness, pitched against the certainty of the science. Tyreman concludes that phronesis adds a corrective dimension to the perception that medicine is a technē. Waring’s paper entitled “Why the practice of medicine is not a phronetic activity” would appear to be a rebuke to Tyreman and others. In fact, it is a critique of Aristotle, who, Waring (2000) argues, felt that medicine was a technē (skill) rather than phronesis. Waring also suggests that a modern-day interpretation of phronesis may work as a conceptual model (moral prosthetic), but this is arguably a deviation from Aristotle’s original notion.

In 2003, Svenaeus suggested that phronesis shows a way forward for medical ethics, given that standard medical ethics courses fixate on rules-based ethics rather than virtue ethics, where phronesis is placed. Phronesis is a deliberative process rather than an outcome. He focuses on Gadamer’s Aristotle-inspired work, observing that the doctor-patient relationship is interpretive, whereby a story about ill health needs to be scrutinized and prioritized toward an agreed best outcome. In other words, phronesis is the best way to apply ethical principles to medical decision-making. Fuks, Brawer, and Boudreau (2012) likewise stipulate that cognition and character are expressed as a synthesis through the concept of phronesis, and that doctoring cannot be simply described by the accumulation of skills that is technē.

Other scholars like LeBlond (2013) write phronesis in terms of the *ends of medicine* and *ways of knowing* (2013). He suggests that the knowledge process moves from *knowing what* (episteme/technē) to *knowing how* (phronesis). George et al. (2023) suggest phronesis is beyond mere competency, stating that “an emphasis on purpose and character within professional reflection promotes excellence, rather than just competency, and engages with what motivated most doctors to enter medicine.”

In total, 15 papers were analyzed to unravel this theme. Only one paper offered critical commentary regarding the adoption of phronesis in understanding medical reasoning, and this comes from Bontemps-Hommen, Baart, and Vosman (2019). They speak of phronesis as a heuristic concept that can be adapted. They define the good of medicine as only known retrospectively, which can be problematic when it comes to decision-making.

### Theme 2—Judgement Versus EBM

One of the dominant dialogues when considering the literature on phronesis in medicine is the contrast between its prevailing EBM or scientism and a medicine that is dependent on exercising judgement in a contextual way, i.e., using phronesis. Thirteen papers emphasized the limitation of scientism and technical rationality. Commentary by Goldner and Bilsker (1995), Fugelli (1998), Muench (2018), and Vidaeff, Turrentine, and Belfort (2022) echos the same sentiment.

Evidence-based medicine remains an essential prerequisite but ultimately, only the practitioner’s clinical expertise, knowledge and practical wisdom will provide the ability to apply general rules of evidence to particular clinical situations. (Vidaeff, Turrentine, and Belfort, 2022)

The key discourses favoring phronesis over technical rationality and EBM are that medicine is messy and complex (Schei, Fuks, and Boudreau, 2019), patient narratives do not fit neatly into the evidence (Montgomery, 2009), scientific rationality is haphazardly applied to individual treatments (Phillips and Hall, 2013), medical judgments are fundamentally about the management of uncertainty (Tyreman, 2000; Muench, 2018), and medicine is constantly evolving regarding medical, technical, and ethical considerations (Hutson and Myers, 1999). Experienced doctors are more likely to rely on judgment, while early career doctors rely more on rules and EBM (Arthur et al., 2015).

The literature goes on to state that outcomes are not always neatly quantifiable and objective (Muench, 2018). EBM can obscure evidence (Macnaughton, 1998). Fugelli expands on the dangers of overdependence on EBM:

The general practitioner must be a master of pragmatic medicine. Rationality, the dominant modern trend, may be dangerous for patients and doctors: (1) advances in technology can give patients and doctors the illusion of mastering the universe; (2) patients complain of being treated like biomachines, without human touch. (Fugelli, 1998, 184)

This is further exacerbated by the commercialization and bureaucratization of medicine, and in this situation, medicine loses its purpose and meaning (Paes, 2019). The concern is that there is an instrumentalist misapplication of reflection (Schei, Fuks, and Boudreau, 2019) and that scientific maxims do not consider philosophical enquiry (Tyreman, 2000; Schei, Fuks, and Boudreau, 2019). For example, Fugelli (1998) suggests that EBM is essential but not sufficient in medical practice. What is really happening is a fusion of rational and emotional decision-making, best represented by phronesis (Russell and Greenhalgh, 2014). Doctors are pragmatists in that they act for the sake of patient well-being and have one key instrument to do this—phronesis (Hutson and Myers, 1999). Doctors use practical reason to navigate the course of illness and disease (Gatens-Robinson, 1986) with interpretation, deliberation, and context being key. Montgomery (2009) refers to this as analogical interpretation and contrasts this with the hypothetico-deductive methods of technical rationality and scientism.

These debates are not new. They caused controversy at the time of the Enlightenment period with Descartes (a Renaissance rationalist) favoring empiricism, technical rationality, and certainty (A.D. 1637). Michel de Montaigne was classed as a Renaissance humanist and contested Descartes. He stressed the importance of meaning, context, and metaphysics. History has instructed us of the dominant scientific rationality narrative that prevails in health care to this day (Muench, 2018).

### Theme 3—Phronesis in the Context of Virtue Ethics

A detailed discourse has been covered by moral philosophers in this publication.

### Theme 4—Working with Phronesis as a Conceptual Model: Problematics and Positionings

The term phronesis is used in different ways by different authors (theme 1). This means it can be difficult to use phronesis as a conceptual framework. Kristjánsson (2015) sought to present an analytical framework that would allow phronesis researchers to identify their own position and identify the conception of phronesis that they are using. This thoughtful paper appeals to all researchers to take a stand on the controversies by presenting four key binaries: universalist or relativist, generalist or particularist, natural or painful, and MacIntyre or Aristotle. In brief, the first binary is socio-moral, and it asks researchers to commit to being universalist or relativist in their conceptions of phronesis. The second binary relates to epistemology and the nature of knowledge. The third binary relates to the psychology of how difficult phronesis is to learn, develop, and master. The final binary is based on the understanding of praxis (Aristotle) or practice (MacIntyre).

Considering this literature review as a corpus, most papers refer to the MacIntyrean concept of phronesis with few staying faithful to Aristotle. This would align with Kristjánsson’s exercise on analyzing four papers with his binaries (Beresford, 1996; Hutson and Myers, 1999; Tyreman, 2000; Kaldjian, 2010). All four were found to be MacIntyrean in their application of phronesis in practice. In the flow of a clinical consultation, the clinician does not intentionally delineate what constitutes an ethical decision, what is a political decision, what is a social decision, and what is a therapeutic decision. Rather, they are considered together in a holistic “all encompassing” way. Grasping Kristjánsson’s binaries thus allows a more nuanced understanding of phronesis.

### Theme 5—Phronesis Orientated Towards Professionalism or for Ethical Decision-Making?

Kristjánsson’s pivotal paper on how to appraise the conceptions of phronesis was used as the screening lens of whether the authors were taking a view of phronesis that was orientated toward the whole of professional practice (MacIntyrean), or a view that focused on ethical decision-making in the moral sphere (Aristotelian). Within this literature review, I found that many authors do not recognize that there is more than one position, and it is only by critically analyzing the papers that their positions become clear.

Toon, in his book *A Flourishing Practice* (2014), applies the work of MacIntyre (1981) to the current state of healthcare provision in the United Kingdom. Being MacIntyrean, Toon takes a view that phronesis is orientated to the whole of professional practice. This is a view also taken by clinician authors such as Sadler, who reviewed professional practice in psychiatry training (Radden and Sadler, 2008), Hilton and Slotnick (2005) who wrote about proto-professionalism, Kinghorn (2010) who wrote about the professionalism movement where professionalism is inseparable from other domains of practice, and Boudreau, Creuss, Cruess, and Fuks, who write about professional identity and physicianship (Boudreau, Cruess, and Cruess, 2011; Boudreau and Fuks, 2015). Hovdenak and Wiese (2018) designed and evaluated a phronesis-orientated undergraduate MBChB curriculum revision, with a view to focusing on professional development. They also take the position that phronesis relates to the whole of professional practice.

Non-clinical commentators seemed more likely to adopt an Aristotelian position, suggesting that phronesis is restricted to ethical decision-making (Schultz and Flasher, 2011; Kristjánsson, 2015; Malik, Conroy, and Turner, 2020). Most work in medicine has a moral dimension as it involves another person and a subjective interpretation of their story; the practitioners do not pause to differentiate the ethical components from everything else. Thus, the literature suggests delineation is not practically relevant. The appraised literature favors a position that covers the whole of clinical practice (MacIntyrean).

### Theme 6—Empirical Understanding of Phronesis

**Fig. 2.**
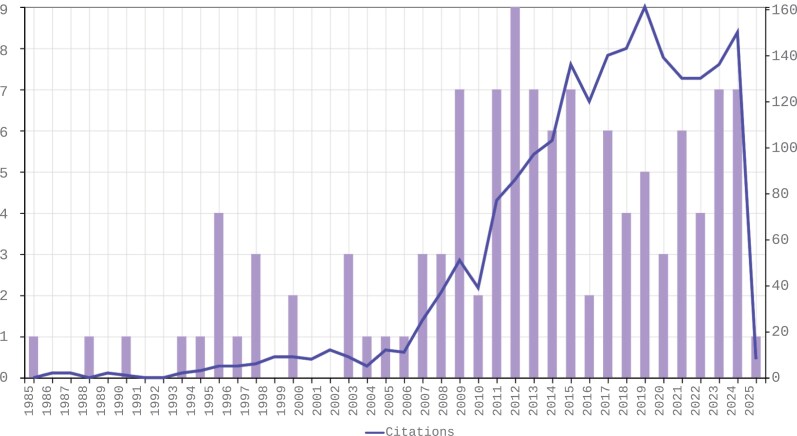
Trends in publications for Phronesis in Medicine (taken from the Web of Science database, accessed December 10, 2024).

As depicted in fig. 2, academic publications on phronesis have grown in the last decade, but this is a relatively recent phenomenon. Much of this work, moreover, has been theoretical in nature. Since 2019, there has been the beginnings of an upturn in empirical work, potentially allowing a more nuanced grasp of phronesis in practice. It is an opportunity to empirically test the viability of these theories in practice and their consistency against real experience (Kotzee, Paton, and Conroy, 2016; Darnell et al., 2019).

More empirical research is needed to make the philosophical concept of practical wisdom better manageable for clinical practices and to gain better understanding of the figurations that elicit or obstruct its manifestation. (Bontemps-Hommen, Vosman, and Baart, 2020b)

Most academic research has the challenge of turning theoretical ideas into practice, and indeed, the theory-practice gap has been widely recognized in relation to phronesis (McKie et al., 2012; Kotzee, Paton, and Conroy, 2016; Chin-Yee, Messinger, and Young, 2019; Darnell et al., 2019; Kristjánsson et al., 2020). This gap in the literature provides an opportunity for empirical work to be conducted. In promising recent moves, the full thesis on *Enacted Practical Wisdom in General Practitioners* is available online (Jameel, 2021) and other empirical work on phronesis in medicine has been conducted and published in well-respected journals (Bodreau et al., 2024; Garcia-Cruz, Garcia-Cruz and Baranda-Tovar, 2023; Kaldjian et al., 2023).

### Theme 7—Individual Phronesis within Organizations

The bulk of the literature focuses on the individual development and attainment of phronesis, but there is some interesting literature emerging on how individual phronesis relates to organizations and institutions. This embraces the concept that an aspiration to eudaimonia would include the communities, societies, and populations over which organizations have influence.

#### Individual phronesis

Regarding nurturing phronesis in the individual, Dunne (2009) describes phronesis as *being,* and technē as *doing*, with phronesis involving a moral imperative. Dunne presents the idea of internal goods of a practice (Bondi et al., 2011). He states these qualities together focus and direct the practitioners’ energy and attention. Extending these internal loci, Svenaeus (2014) speaks about *feeling* and Malik, Conroy, and Turner (2020) write about the importance of *motivation* as a key instigator of phronesis. Saraga, Boudreau, and Fuks (2019) along with Kotzee, Paton, and Conroy (2016) speak of the importance of personal *engagement* in the task, with Saraga using the term engagement synonymously with phronesis and Kumagai (2014) linking personal *agency* to phronesis.

Highlighting a different concern, Bishop and Rees allude to the importance of measurement in assessing wisdom (referring to prosocial behavior). Now measurement plays into the technical rationalist thinking, and, as Dunne argues, wisdom development in terms of feelings and motivation may be hard to measure (Bondi et al., 2011). Muench brings in a further interesting perspective regarding the circumstances in which phronesis can thrive, suggesting that *context* can affect the embodiment of phronesis. He describes how third-world countries with sparse medical resources would require a more rapid embodiment of phronesis (Muench, 2018) because the rationing of resources in any setting may fast track the development of the adjudicative function of phronemos.

#### Phronesis and organizations

Russell and Greenhalgh (2014) looked at how decisions were made on Health Commissioning Group funding research panels. They concluded that rational universal rules and policies were a front for control, order, and accountability. Decisions often boiled down to individual instinct and empathy, which led to convincing the panel to act a certain way. The authors conclude that decisions in these panels were guided by *collective phronesis*, identifying a combination of the subjective and the rational aspects of the deliberation. Along similar lines, Davidoff (2011) spoke of phronesis being needed in healthcare improvement endeavors, while Mugerauer (2021) asserts that phronesis is especially important in regard to public decision-making. Stanak (2019) outlines tragedy in the neonatal setting, describing the uncertainty of the work and moral injury as a result of difficult decisions, and argues that healthcare organizations need to recognize these ethical issues and their ramifications. He calls for organizations to understand phronesis so that better decisions can be made.

In light of the collective dimensions of decision-making, other scholars like Bontemps-Hommen, Baart, and Vosman (2019) propose a new definition of practical wisdom, which can be found in theme 9. Their definition speaks of acting jointly, acknowledging institutional and systemic pressure, and working in circumstances of complexity, implying the need for shared goals and organizational leadership. Social practices need to be included in this definition if it is to truly attend to flourishing, and this is more forcefully stated in a paper by Martimianakis (2016) on medical education, social responsibility, and praxis.

For context, Aristotle’s *Nicomachean Ethics* only really alludes to individual phronesis. In a business ethics paper, Kristjánsson outlines a conceptualization of collective phronesis taken from Aristotle’s *Politics*. Here, Aristotle describes phronetic rulers contending with civic virtues at the state level (usually city-states about the magnitude of medium-sized businesses). Kristjánsson (2022) endorses the practical translation of Aristotle’s ideas through Ikujiro Nonanka’s thinking on leadership and knowledge flow within industry. Phronesis needs to be understood and enacted at an organizational level, and this includes healthcare organizations.

### Theme 8—Telos, Patient Care, Narrative, and Ways of Knowing

Scientific ways of knowing are necessary and important, but I wish to focus on the neglected ways of knowing, which the literature on phronesis captures. The literature suggests that the physician needs to know the science and the world (Kumagai, 2014). Chin-Yee, Messinger, and Young (2019) refer to it as “multiple ways of knowing,” suggesting that we can know the world through *self-awareness* and through the *humanities* (Chin-Yee, Messinger, and Young, 2019; see also Hilton and Slotnick, 2005; Boudreau and Fuks, 2015). One of the most important learning outcomes in engaging with the humanities is the importance of *subjective interpretation*. Here objective truths of scientism fade and the complexity of interpretation and the uncertainty that it brings come to bear. Physicians should accept this, rather than feeling threatened by it, and build it into a heuristic of clinical reasoning (Gatens-Robinson, 1986).

The literature focused particularly on *narrative* as a means to understanding and studying phronesis in doctors (Schultz and Flasher, 2011; Russell and Greenhalgh, 2014; Kotzee, Paton, and Conroy, 2016). Leblond (2013) attempts to describe what needs to happen next in progress toward phronesis from a narrative. Intuition was also mentioned as a way-of-knowing (Montgomery, 2009; Braude, 2013; Russell and Greenhalgh, 2014). Intuition is not simply premonition; it is related to experience and expertise via tacit knowledge. Good medicine should also recognize that decision-making is a co-construction between doctor and patient who identify shared goals (Reeve, 2010; Schultz and Flasher, 2011).

## IV. THE FUTURE

### Theme 9—Theory to Practice, Knowledge Translation, and Decision-Making

Medicine is both a natural science and a human science. To perform well in medicine, the deductive aspects need to be paired with the interpretive aspects. Bontemps-Hommen et al. have produced some fascinating research papers seeking to determine practical, wise decisions in clinical settings (Bontemps-Hommen, Vosman, and Baart, 2020a, 2020b). This has resulted in them devising a contemporary, holistic definition of practical wisdom, which is founded in Aristotelian phronesis but acknowledges the current complexity of medicine and the recognition that phronesis is not a solely individual pursuit (theme 7). Consequently, we define practical wisdom as:

The capability, which emerges in acting jointly within medical practices, of knowing how to remain focused on achieving the good for every individual patient, in ever‐changing situations, within the context of the practice and its telos, and of how to accomplish this by the most appropriate means, while dealing with complexity and institutional and systemic pressure. (Bontemps-Hommen, Vosman, and Baart, 2020b)

Dieppe explores phronesis in orthopedic surgery (knee joint replacements) where every patient encounter requires decision-making based on wisdom, experience, and scientific data. He points out that pain cannot be measured and therefore can only be subject to interpretation. Theories and generalized rules cannot be applied blindly because each encounter is unique, and the context requires interpretation (Dieppe, 2011). Importantly, Goodyear-Smith (2011) adds the caveat that *we cannot know everything about context*, and physicians often only know what the patient chooses to tell us.

The most rigorous analysis of the theory to practice problem, and knowledge translation, is provided by Greenhalgh and Wieringa (2011). They explore what has happened in Medicine (United Kingdom) and argue that the term “knowledge transfer” has become constraining. This is due to policymakers and management leaders using (theoretical) evidence obstinately. Greenhalgh and Wieringa contrast this to the disciplines of philosophy and sociology, where knowledge is created, constructed, and embodied, and where it is presumed to be value-laden and is less obstinately used. Greenhalgh and Wieringa conclude by offering four recommendations in bridging the knowledge-action gap: the need for strategic-level approaches in the cycle of development, implementation, and revision of clinical guidelines in a way that recognizes and captures phronesis. This links in with theme 7 above.

### Theme 10—Phronesis for Dealing with Complexity and Moral Distress

The presence of ethical deliberation is more pronounced in situations of tragedy (Stanak, 2019). Stanak discusses grey area decisions and moral injury where scientific frameworks do not seem to capture or address the complexity. Uncertainty and complexity are inherent to many clinical situations (Tyreman, 2000; McKie et al., 2012; Bontemps-Hommen, Baart, and Vosman, 2019), but the ethical frameworks of deontological principlism are distressingly arbitrary in dealing with complexity. Hall (2011) observes, for example, that principles are often misused to justify questionable innovations. Illustrating this insight, Hutson and Myers discuss the complexity of dealing with a baby with profound disabilities from their clinical perspective of pediatric urology and thoracic surgery, which requires deliberation in medical, technical, and ethical considerations. These considerations have a wide array of ramifications affecting the baby, the family, and society as a whole. The authors therefore present phronesis as a viable intellectual tool in such circumstances. Kinghorn (2010) similarly refers to phronesis as supportive in such circumstances. Bontemps-Hommen, Baart, and Vosman (2019) describe it as a helpful heuristic concept in dealing with complexity.

One paper by Ko et al. (2020) observed the complexity and challenges in the nursing profession. Ko’s team specifically explored moral distress, defined as not being able to carry out their moral intention. They analyzed the nurse’s actions rather than the cause of moral distress. The focus was on reframing and developing their phronesis (Ko et al., 2020). The process described in Ko’s paper is not dissimilar to the psychoanalytic approach that is performed in Balint groups. Balint groups are an opportunity for clinicians to reflect on psychodynamic factors in relation to patient care. The goal in these groups is not a clinical solution but a creative insight into the relationship and the causes of moral distress. Muench (2018) made a connection between Balint groups and the development of phronesis. Similarly, a study led by Professor Plews-Ogan (2016) observed what helped develop wisdom in doctors who have made medical errors. Plews-Ogan and Sharpe have written about phronesis in medical practice. They speak about the decisions we must make in complex, ambiguous, and contradictory circumstances where information is often incomplete, and they contextualize this with institutional case studies (Plews-Ogan and Sharpe, 2022).

### Theme 11—How to Develop Phronesis

The predominant narrative within the literature refers to how to develop phronesis in the individual. I also discuss how organizations (educational and healthcare systems) need to promote the development of phronesis, and class this collective dimension as being outside of the individual. Attempts to develop phronesis are subject to erosive influences and these are explored as well.

Kinghorn (2010) along with Hilton and Slotnick (2005) have explicitly aligned the development of phronesis with *professional development*. There are some clear parallels in the attributes and mental habits required in developing both. Kumagai (2014) speaks about the clinical learner realizing their position in the world as an actor and realizing their responsibility and accountability in addressing human needs and values. To appreciate this fully, clinical learners need to understand the *goals of medicine* (personal and societal), and this has been reinforced by a few authors (Frank, 2004; Baum-Baicker and Sisti, 2012). With a goal in mind, a sense of *agency* can develop (Kumagai, 2014) and therefore the *motivation* to act (Baum-Baicker and Sisti, 2012; Kumagai, 2014).

A key to developing phronesis is *self-awareness* (Hilton and Slotnick, 2005; Bishop and Rees, 2007; Baum-Baicker and Sisti, 2012; Muench, 2018; Paes, Leat, and Stewart, 2019). This self-awareness helps the learner address their misperceptions, have insight into ways-of-knowing, and develop the *intellectual humility* to want to know more about what is relevant to the context. Baum-Baicker and Sisti (2012) write about the importance of *emotional balance*, which is also an attribute of self-awareness and effective reflection. Central to self-awareness is *reflective practice*, which has been identified as a key component in nurturing phronesis (Baum-Baicker and Sisti, 2012; Kumagai, 2014; Muench, 2018; Schei, Fuks, and Boudreau, 2019). It promotes discourse on ill-structured problems, accepting paradox as inevitable. The ideal way to reflect is via case-based discussion (Muench, 2018) involving exploratory dialogue (Chin-Yee, Messinger, and Young, 2019).

*Apprenticeship, mentorship, and role models* are connected themes that allude to how reflective learning is embedded. Carnevale describes how highly contemplative experiential learning through apprenticeship is essential in developing phronesis. He suggests that morally meaningful learning is drawn out through dialogue with a *supportive supervisor* (Carnevale, 2007). Moral meaning has also been highlighted by Frank (2004) and Muench (2018) where the mentor or teacher should be someone who embodies phronesis, for moral meaning [phronesis?] to develop effectively. Role models and clinical teachers can inspire habituation of virtue in the learner; the learner comes to learn more about role morality (Hilton and Slotnick, 2005; Radden and Sadler, 2008). There is consensus that *experience* along with reflection-on-experience is a key aspect of developing phronesis. Chin-Yee introduces the concept of *slow medicine*, which is intentional deliberation and reflection (Chin-Yee, Messinger, and Young, 2019).

A number of authors have suggested that *understanding narrative* is important in developing medical phronesis (Baum-Baicker and Sisti, 2012; Chin-Yee, Messinger, and Young, 2019; Paes, Leat, and Stewart, 2019), and some extend this to a wider appreciation of the *humanities* and its role in developing phronesis (Boudreau and Fuks, 2015; Chiavaroli and Trumble, 2018).

Developing phronesis in the individual involves *organizational and institutional support*. For clinicians in training, this would include their academic institutions and the healthcare organizations, where the apprenticeships take place. There needs to be organizational *commitment to moral development* (Baum-Baicker and Sisti, 2012; Paes, Leat, and Stewart, 2019; Bontemps-Hommen, Vosman, and Baart, 2020a; Mugerauer, 2021). Curricula need to name explicitly the need for phronesis development, and this happens through experiential learning (Leathard and Cook, 2009). This should be *incorporated into curriculum design* with explicit discussion about the *goals of medicine* (Frank, 2004; Baum-Baicker and Sisti, 2012), understanding the different ways of knowing in medicine and promoting phronesis as an executor in decision-making processes (Leathard and Cook, 2009; Chin-Yee, Messinger, and Young, 2019). Promotion of prosocial behavior is also suggested (Bishop and Rees, 2007).

Hilton and Slotnick (2005) describe six domains of professionalism that develop using phronesis, and they refer to this development as proto-professionalism. Paes, Leat, and Stewart (2019) empirically derived six internal and six external influences in developing practical wisdom. It appears that the Paes, Leat, and Stewart intervention areas could be subsumed in the Hilton and Slotnick categories.

Many of the authors cited in this section refer to aspects that erode the development of phronesis. These setbacks include the hidden curriculum (Hilton and Slotnick, 2005), anti-mentors, which can both promote and inhibit phronesis (Baum-Baicker and Sisti, 2012), unhelpful pedagogies (George, Urch, and Cribb, 2023) and the dominance of assessment and competency-based education driving learning (McGee, 1996). The harshness of the healthcare system, environmental stress, commercialization, and technical rationality applied unreflectively have also been noted (Fugelli, 1998), while another paper has suggested that practical wisdom cannot develop in constraining systems (Bontemps-Hommen, Vosman, and Baart, 2020a).

If medical educators and healthcare leaders want to develop practical wise clinicians, they need to be aware of the issues outlined in this section. Despite assertions suggesting phronesis can be taught (e.g., Pellegrino and Thomasma, 1993), the later authors, who explore more nuance, concur that phronesis cannot be taught. They argue that educators can only set the conditions and provide the opportunities for it to develop. This aligns with Kemmis (2012) who speaks of phronesis more generally in relation to professional practice.

Phronesis is not something that can be taught: it can only be learned, and then only by experience. To the extent that phronesis can be taught at all, it can only be taught indirectly. (Kemmis, 2012, 148)

### Theme 12—Phronesis Education

As noted, some authors argue that phronesis cannot be taught (Gunasekara, Patterson, and Scott, 2017; Chiavaroli and Trumble, 2018; Muench, 2018). However, earlier authors have suggested that it can be taught, and Kinghorn takes a strong Aristotelian stance on this point (Kinghorn, 2010). Clinicians, who have written about phronesis, put forward a strong call for phronesis education, but often have no proposal for how this should be done (Paes, 2019; Elledge et al., 2020). Chiavaroli and Trumble have said that phronesis provides theoretical and pedagogical legitimacy for embedding wise judgment, moral reasoning, and contextual awareness into medical curricula (2018). More recently, Bontemps-Hommen et al. have subsequently elucidated what can be taught in relation to developing phronesis in clinical practice (Bontemps-Hommen, Vosman, and Baart, 2020a, 2020b).

Character-based educational interventions can have a positive influence on professional practice, but introducing them into professional education may not in itself be sufficient for the development of phronesis, which requires institutional and societal investment (Harrison and Khatoon, 2018). For example, Boudreau, Cruess, and Cruess (2011) consider if students should be recruited to medical programs on the basis that they have a good moral foundation for phronesis.

The first step in considering phronesis education is *raising awareness of its importance*. The term is unfamiliar to medical educators, and its adjudicative function and importance in medical decision-making are not widely known. Scholars like Dowie (2000) have called for phronesis to be in the working vocabulary of all medical educators. However, a practical obstacle to this could be the philosophical and theoretical baggage associated with the ancient terminology. Jobbing clinicians want succinct clarity regarding impact and relevance.

The next question is, how could phronesis be *embedded in the curriculum*, and should it be embedded in a curriculum? If phronesis is value-laden, and most values are learned via the hidden curriculum (Hafferty and Franks, 1994), the educators need to appreciate that phronesis, and the values that underpin phronesis, are learned both ways. Chiavaroli and Trumble (2018) state that medical educators can learn from humanities educators as they set the conditions for which more tacit learning happens. Dieppe (2011) calls for educationalists to ensure the curricula embody open pluralism, engaging communities outside of medicine to help medical educators understand better. Dowie (2000) articulates that curricula need to be able to encompass wisdom concepts, including the limits of knowledge.

Ethical practice and moral development are the subject of much theorizing and criticism in the body of literature reviewed. Fugelli (1998) criticized the political and cultural factors that have eroded the humanity of clinical practice. Other authors have called for attention to moral development, and in particular, the use of a *virtue approach* (Gillies, 2005; Radden and Sadler, 2008; Baum-Baicker and Sisti, 2012; George, Urch, and Cribb, 2023). In these ways, the literature critically reflects on *how medical ethics is currently taught*, treating it as a central domain in which to understand phronesis. Many authors were disappointed in the principlism or rules-based approach to ethical issues, which can seem somewhat removed from the goals of medicine and understanding “the good” (Dowie, 2000; Dieppe, 2011; Hall, 2011). A phronetic approach to formation requires the learner to be reflexive and integrate their moral grounding with the professional virtues and values expected of the profession. Hilton and Slotnick (2005) state that not adopting and creating conditions for a phronesis approach will lead to having cynical clinicians who become disillusioned due to a lack of support and poor environmental conditions, i.e., not flourishing, which would align with Aristotle’s notion that phronesis leads to flourishing.

McKie et al. (2012) describe three erosive influences on the holistic integration of phronesis in nursing education. These include the *modularization* of higher education, the *uncoupling of pastoral and academic support,* and the *theory-practice gap*. Hilton and Slotnick (2005) write about the deformative experience created by the hidden curriculum, acknowledging that *environmental stress* inhibits the development of phronesis. Institutions and organizations should be mindful of this and should mitigate the environmental erosions where possible. Hall (2011) suggests communities of practice with phronesis as a goal might be a way forward. Paes (2019) has likewise alluded to the promotion of learner self-efficacy (believing they can succeed) and relational agency (learning how to gain help and give help) as enablers to developing phronesis.

*Assessing phronesis* is a contentious issue. Some authors feel assessing it is imperative if phronesis is to be taken seriously. Hall (2011) sees this as a very positive step in the development and assessment of phronesis. Boudreau, Cruess, and Cruess (2011) have also introduced a summative P-MEX (work-place-based assessment) to assess aspects of phronesis. Kumagai (2014) asserts that competency-based assessment erodes the development of phronesis, and he calls for attention to quality, not just competence. George suggests virtue ethics provides a better framework for developing phronesis, rather than competency models (George, Urch, and Cribb, 2023). This is echoed by Paes (2019) who contends that medical education is mechanistic and systems driven, increasingly losing touch with the purpose of producing good doctors, which then translates into the assessments. Dowie (2000) takes a pragmatic stance, suggesting that competence needs to be assessed summatively, but the aspect of phronesis that involves the quality of the clinician-learner should be formatively assessed.

## V. LITERATURE REVIEW SYNTHESIS

The 12 themes from the literature review have now been presented (see table 1 for a summary statement of themes). In conclusion, I can now synthesize some of the main concepts, addressing the research question, “*What narrative commentary prevails in relation to Phronesis in Medicine*?”

Given the ways in which the concept of phronesis has evolved from its Aristotelian origins, its application to medical practice conceptually has been understood in different ways over the years by different authors. For example, there are two factions with regard to what phronesis does and when it is useful. One uses phronesis synonymously with professionalism and the whole of professional practice (MacIntyrean view), while the other describes it in the context of ethical decision-making only (Aristotelian view). Nonetheless, there is wide agreement about the idea that phronesis is a process and not an output. It identifies the morally salient issues by interpretation in a particular context and can be particularly helpful in dealing with complexity, moral injury, or distress. Or put differently, phronesis is not simply enactment, which suggests a visible *doing* with an output. It is best defined as being embodied (*doing, being,* and *feeling*) which includes the doctor’s motivation and internal activity.

To be sure, phronesis discourses have largely focused on the individual. There is more published work on the character and moral formation of individuals than of organizations. However, there is a small but growing literature on how phronesis fits into organizational decision-making. In order to further its practical impact, organizational and institutional appreciation of phronesis may be an important area of growth for this literature.

The epistemology (theory of knowledge) and ways of knowing in relation to clinical practice are particularly important. This is often juxtaposed against the current predominance of technical rationality. There is much work to be done in convincing the technical rationalists and empiricists about the value of critical theory and constructivist approaches to knowing. These are also essential in the practice of medicine, which is seen as an interpretive task by many authors on phronesis. Phronesis thus has the potential to fill a theory-practice gap, with its moral orientation as a guiding force. There is a lack of empirical work that could illuminate the theoretical questions relating to phronesis. The last 10 years have seen a slow increase in empirical work compared to most prior publications, which were purely theoretical.

In terms of the application of phronesis in education, some authors feel that it cannot be taught. Those that suggest it can emphasize self-awareness and habits of the mind, reflective practice teamed with experience and mentorship, role models and exemplars, habituation, and the role of the humanities and clinical reasoning and decision-making processes. There is some consensus that phronesis requires cognitive, reflective, and affective synergy in the individual. However, there is also a strong awareness of the significant obstacles to developing phronesis. In higher education, these include the hidden curriculum, modularization, poor faculty awareness, assessment, environmental stress, and ethics education, specifically the uncoupling of ethical principles from context and reliance on rules-based ethical frameworks, rather than a focus on how virtue ethics can assist.
